# A New Frontier for Drug-Coated Balloons: Treatment of “De Novo” Stenosis in Large Vessel Coronary Artery Disease

**DOI:** 10.3390/jcm13051320

**Published:** 2024-02-26

**Authors:** Alessandro Sciahbasi, Tiziano Maria Mazza, Chiara Pidone, Simona Samperi, Edoardo Cittadini, Antonino Granatelli

**Affiliations:** Interventional Cardiology, Sandro Pertini Hospital—ASL RM2, 00157 Rome, Italy; tiziano.mazza@gmail.com (T.M.M.); chiara.pidone22@gmail.com (C.P.); simona.samperi@aslroma2.it (S.S.); edoardocittadini@yahoo.it (E.C.); antonino.granatelli@aslroma2.it (A.G.)

**Keywords:** drug-coated balloon, percutaneous coronary interventions, paclitaxel, sirolimus

## Abstract

**Background**: Drug-coated balloons (DCB) are a well-established option for treating in-stent restenosis endorsed by European Guidelines on myocardial revascularization. However, in recent years, a strategy of “leaving nothing behind” with DCB in de novo coronary stenosis has emerged as an appealing approach. **Methods**: We performed a systematic review to evaluate the current literature on the use of drug-coated balloons in the treatment of de novo stenosis in large vessel disease. **Results**: Observational studies, as well as randomized studies, demonstrated the safety of DCB percutaneous coronary interventions (PCI) in large vessel disease. The rate of major adverse cardiac events is even lower compared to drug-eluting stents in stable coronary artery disease. **Conclusions**: DCB PCI is feasible in large vessel disease, and future large, randomized studies are ongoing to confirm these results.

## 1. Introduction

The advent of drug-eluting stent (DES) revolutionized the history of percutaneous coronary interventions (PCI) reducing the rate of abrupt vessel closure after plain balloon angioplasty, lowering the rate of coronary restenosis, and improving the outcome of patients with coronary artery disease [1]. Nowadays, DES is considered the default treatment for PCI, but its use presents some limitations: a permanent metal prosthesis left in the vessel wall prevents regular vasomotion of the artery, and the use of metal scaffolding and polymers increases the risk of late and very late stent thrombosis [2]. In particular, the rigid stent prevents relaxation and constriction of the artery, impairing endothelium-dependent vasodilation [3], which is a marker of poor outcome [4].

In recent years, drug-coated balloons (DCB) have emerged as a potential alternative to DES, combining the advantage of local drug release during balloon angioplasty without the long-term disadvantage of DES in terms of impaired vessel motility and increased risk of late thrombosis [5]. The use of DCB delivers a high concentration of antiproliferative drug to a target lesion, and the drug is rapidly transferred to the entire vessel wall. It also decreases chronic inflammation (due to the long-term persistence of polymers) and helps maintain coronaries’ original anatomy, especially in cases of bifurcation, tortuous, or small vessels. A DCB procedure leaving no metal in the arterial wall can reduce vessel thrombogenicity, allowing a de-escalation of dual antiplatelet therapy in patients with higher bleeding risks. On the other hand, the use of DCB has some limitations: the elastic recoil of the vessel wall after balloon angioplasty, the risk of major vessel dissections, arterial remodeling, and individual variability of drug response are still issues to be overcome [6]. Moreover, DCB performance is strictly related to the multiple components of this device: the drug, the carrier, the polymer, and the excipients [7], and consequently, a class effect for different DCBs cannot be considered.

Initial studies with DCB in PCI were performed more than 15 years ago [8], but currently, strong evidence for its use is still limited to the treatment of in-stent restenosis [9] and in “de novo” small vessel disease [10,11]. The aim of this review is to provide an overview of the available data and different devices utilized, focusing on a new possible field of use of DCB: the treatment of “de novo” stenosis in large vessel coronary artery disease.

## 2. Drug-Coated Balloon Components

There are many DCBs on the market characterized by a different combination of drugs, carriers, and polymers. Each component of the device is essential to the acute and long-term efficacy of the DCB.

### 2.1. The Drug

Among all the different drugs tested on the DCB, paclitaxel, and sirolimus are those that obtained the best results and are the most utilized.

- Paclitaxel-coated balloons. Paclitaxel is an anti-neoplastic, cytotoxic drug that binds to microtubules in mitosis, inhibiting cell division and proliferation [7]. Paclitaxel is the most widely used drug in the setting of DCB due to its physiochemical properties and, in particular, due to the lipophilicity of the drug that allows it to rapidly penetrate the cell membrane of smooth muscle cells and to support a long-lasting antiproliferative action even after a brief, single-dose application [12]. The drug has a narrow therapeutic window, and the dosage utilized on DCB ranges between 2 and 3.5 µg/mm^2^.
- Sirolimus-coated balloons. Sirolimus is a cytostatic drug that inhibits smooth muscle cell proliferation and migration by blocking cell-cycle progression at the G1/S transition [13]. Compared to Paclitaxel, sirolimus has a wider therapeutic range (1–7 µg/mm^2^), greater anti-restenotic and anti-inflammatory effects, slower tissue absorption, and short tissue retention. However, a major disadvantage of the drug is its non-lipophilic nature, which makes tissue absorption and elution more difficult, requiring a dedicated carrier to allow diffusion and penetration in the vessel wall [14]. Moreover, the drug should be continuously delivered over time, and dedicated technologies are necessary to maintain a constant release.

### 2.2. Carriers and Polymers

Carriers and polymers are, in general, essential to allow the retention of the drug during the vascular transit, to provide adhesion of the drug to the vessel wall during the inflation, and to ensure a rapid and homogenous drug transfer and deposition in the tissue. Because of their bioadhesive surface, these coating materials guarantee an easy penetration into the arterial wall, reduce mechanical trauma, and increase the antiproliferative effectiveness of the drugs used, keeping them adhesive locally into the vessel wall and avoiding drug dilution. For the first time in 2003, Sheller et al. [15] demonstrated that the association of Paclitaxel with ipopromide obtained a complete inhibition of proliferation of smooth muscle cells, and the effect was superior compared to Paclitaxel alone. Since then, for Paclitaxel-coated DCB, other different additives such as urea, Acetyl tributyl citrate, or *n*-Butyryl citrate have been tested with good results [7]. 

For Sirolimus-coated DCB, carriers, and polymers are even more important in order to obtain a prolonged release of the drug after balloon inflation. Dedicated technologies have been tested: nanocarriers in the form of nanoparticles containing Sirolimus [16], spherical micro-reservoirs [17], or crystalline coatings of Sirolilmus [18] are currently the most promising formulations.

## 3. Current Use of DCB

DCBs are a well-established option for the treatment of in-stent restenosis, and European Guidelines on myocardial revascularization endorse their use for the treatment of in-stent restenosis of BMS or DES (Class I, Level A) [19]. As a matter of fact, different studies showed the superiority of a DCB strategy over plain balloon PCI in the treatment of in-stent restenosis [8,20]. Another emerging indication for DCB includes the treatment of “de novo” stenosis in small vessel coronary disease, defined as lesions in vessels ≤2.5 mm in diameter. In the PICCOLETO II study [11], 232 patients were randomized to paclitaxel DCB (118 patients) or DES (114 patients), and at six months follow-up, the DCB arm showed a significantly lower late loss of 0.04 ± 0.28 mm as compared to 0.17 ± 0.39 mm in the DES arm (*p* = 0.03). At three years follow-up, major adverse cardiac events (MACE) and acute vessel occlusion occurred more frequently in the DES group (20.8% vs. 10.8%, *p* = 0.046) compared to DCB (4% vs. 0%, *p* = 0.042). Of note, MACE and acute vessel occlusion occurred more frequently in the DES group (20.8%) compared to the DCB Group (10.8%, *p* = 0.046). 

Few data are available comparing head-to-head Sirolimus and Paclitaxel DCB, but recently, the TRANSFORM I trial compared these two DCB directly in the treatment of small vessel disease. The trial that included only 121 patients (129 lesions) failed to demonstrate the non-inferiority of Sirolimus DCB compared to Paclitaxel DCB in terms of net lumen gain (0.25 ± 0.40 mm with Sirolimus DCB and 0.48 ± 0.37 mm with Paclitaxel DCB (Pnon-inferiority = 0.173), and smaller late loss (0.00 ± 0.32 mm vs. 0.32 ± 0.47 mm; *p* < 0.001) at six months follow-up even though there were no differences in major adverse cardiac events [21]. However, a major limitation of the study is the limited number of patients enrolled, and further studies are necessary to confirm these results. 

## 4. A New Frontier: Large Vessel Coronary Artery Disease

Based on the encouraging results reported for the use of DCB in small-vessel disease, continuous efforts have been made to investigate the role of this promising technology in large native vessels. There is no generally accepted definition of large vessels in the literature, and in our subsequent review, we chose a cut-off of 2.75 mm to define large coronary arteries as documented in previous studies [11,22,23]. Most of the feasibility data of DCB PCI in large vessels come from sub-analysis of larger observational retrospective or prospective studies with few dedicated studies. Data obtained from observational studies [24,25,26,27,28,29,30,31] are summarized in Table 1, whereas randomized studies comparing DCB and stent are presented in Table 2 and Table 3 [32,33,34,35,36,37,38].

In a large retrospective study conducted by Yu et al. [25], over a total of 595 de novo lesions treated using DCB, 222 lesions were in large vessels (diameter greater than 2.8 mm). Over an average clinical follow-up period of 10.1 months, the large vessel group had no MACE nor target lesion revascularization. Subsequently, Rosenberg et al. [23] analyzed data from the international, multicenter DCB-only registry that prospectively enrolled patients in 8 European and 6 Malaysian centers treated with paclitaxel-iopromid-coated DCB: of the 686 patients with de novo lesions, 234 were propensity matched stratified into small and large vessel coronary artery disease (cut off ≥2.75 mm). The study confirmed that DCB-only PCI in large coronary arteries with de novo lesions was safe with a 6.1% rate of MACE that was similar to that in the small vessel group (5.7%, *p* = 0.903) despite almost half of the included coronary lesions were considered as complex. Probably, the low event rate in the study is mostly explained by the strict compliance of all investigators with predilation (more than 90% of procedures) using uncoated balloons having a balloon to-vessel ratio of 0.8–1.0. Moreover, the length of the DCB was chosen to be at least 2 to 3 mm longer than the lesion ends, and DCB PCI was performed only in the absence of flow-limiting dissections (C–E) or significant recoil.

**Figure 1:**
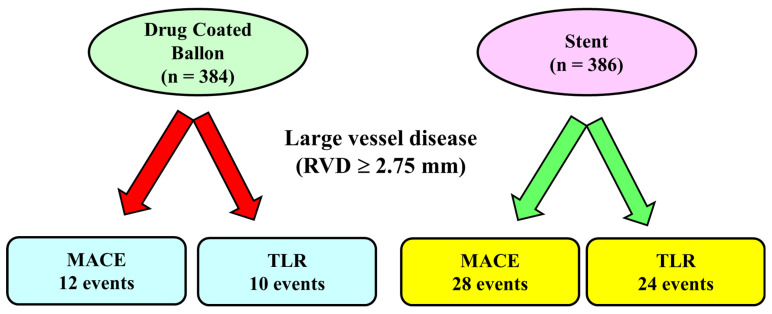
Main results of a meta-analysis of 7 randomized controlled trials in large vessel coronary disease.In this meta-analysis, including lesions with reference vessel diameter ≥ 2.75 mm, the number of major adverse cardiac events and the rate of target vessel revascularization was lower in the Drug-coated Balloon Group compared to the stent Group. MACE: Major adverse cardiac events, TLR: Target Lesion Revascularization.

The largest randomized trial including large vessels is the DEBUT Trial [34], which included 208 patients (83% with vessel diameter > 2.75 mm) assigned to DCB PCI (*n* = 102) or bare metal stent (*n* = 106). Exclusion criteria were in-stent restenosis, flow-limiting dissection or substantial recoil (>30%) of the target lesion after predilation. At 9 months, MACE had occurred in one patient (1%) in the DCB group and in 15 patients (14%) in the stent group. An important point was that in the stent group, there were two definitive stent thrombosis events, whereas no acute vessel closures were observed in the drug-coated balloon group. Further studies confirmed these results [32]: Nishiyama et al. performed a single-center randomized clinical study including 60 patients (30 randomized to DCB and 30 patients to stents) with stable coronary artery disease, excluding lesions severely calcified, in the left main trunk and chronic total occlusions. No acute coronary syndromes or restenotic lesions were enrolled. There were no significant differences between the two groups in major risk factors such as age, gender, hypertension, dyslipidemia, diabetes mellitus, smoking, chronic kidney disease, and family history of ischemic heart disease. At eight months of follow-up, target lesion revascularization rates were very low and similar in the two groups: zero patients (0.0%) in the DCB group and two (6.1%) in the DES group (*p* = 0.193). It should be noted that the use of intravascular ultrasound before DCB application and the short follow-up probably influenced the results. Recently, a meta-analysis of 7 randomized clinical trials comparing DCB and stents in large de novo coronary artery disease [39] included 770 patients (384 randomized to DCB and 386 to stent). The mean age of patients ranged from 49 to 77 years, with the majority of patients (512 patients, 66%) undergoing PCI for acute coronary syndromes. The length of follow-up ranged from 6 to 12 months, and 248 patients (from 2 different studies) used a bare metal stent as a control. All the DCBs used in the studies included in the meta-analysis were paclitaxel-coated balloons. The study showed that the MACE rate was significantly reduced by 52% in the DCB group compared to the stent group (12 vs. 28 events, *p* = 0.04 respectively, Figure 1). The target lesion revascularization rate was also reduced in the DCB group (10 events) compared to the stent group (24 events). Differently, the rate of myocardial infarction that was reported in 4 trialswas not reduced by the use of DCB compared with stents. Major limitations of this analysis were the small sample size and the use in some studies of bare metal stents rather than DES, which are devices no longer utilized in the majority of the centers. 

Good results of DCB PCI have been observed even in the context of unprotected left main disease: in the SPARTAN LMS [30], 148 patients with left main stenosis have been treated by DCB (41 patients) or DES (107 patients). The 41 patients who were treated with a DCB were all treated with an iopromide paclitaxel DCB, whereas the DES group included Sirolimus, Everolimus, or Zotarolimus eluting stents. Patients treated by the DCB were older compared to the stent group and had more challenging coronary anatomy, including more than 70% of true bifurcation compared to less than 50% in the DES group (*p* = 0.006). DCB procedures were associated with less use of contrast media (mean 144.5 ± 41.3) mL compared with DES procedures (mean 176.5 ± 67.1 mL, *p* = 0.006). After an average follow-up of 33.9 ± 19.9 months, there was no significant difference in all-cause or cardiovascular mortality (4.9% for the DCB group and 6.5% for the DES group, HR 1.21 [0.31–4.67], *p* = 0.786) that was confirmed significant after propensity score-matched analysis [HR: 0.31 (0.05–1.930, *p* = 0.210]. The results of this study, although interesting, are associated with some limitations and potential bias: a retrospective design of the study, a limited number of patients enrolled, and procedures performed in a high-volume, large tertiary referral center. Consequently, larger dedicated and randomized studies are necessary to confirm these results. 

DCB in large vessels has been tested even in the context of acute coronary syndromes (ACS). In the REVELATION trial [36], between October 2014 and November 2017, a total of 120 patients with acute ST-elevation myocardial infarction due to coronary stenosis in large coronary artery were randomized to DES (60 patients) or DCB angioplasty (60 patients) with the Pantera Lux balloon (Biotronik AG, Buelach, Switzerland). There were no significant differences in baseline characteristics between the two groups, with a mean age of 57 years and almost 90% of patients being male. Procedural characteristics, too, were similar between the two groups, with a majority of single vessel disease (more than 70% of patients), and the culprit lesion was located in the right coronary artery in 48% of the patients. In the DCB group, there were 8 cases of coronary artery dissection greater than or equal to type C requiring bailout stenting. At nine months follow-up, there were no significant differences between DCB or DES in terms of late luminal loss (0.05 ± 0.13 mm vs. 0.00 ± 0.05 mm, *p* = 0.51 respectively) or MACE (3% vs. 1%, *p* = 1.00 respectively). In 73 patients (34 in the DCB group and 39 in the stent group) at nine months follow-up, a fractional flow reserve was also measured. The mean FFR was 0.92 ± 0.05 in the DCB Group and 0.91 ± 0.06 in the stent group (*p* = 0.27). Despite these good results of DCB in the setting of ACS, two observational studies [40,41] have raised some concerns due to a higher risk of cardiovascular events associated with the use of DCB. Uskela et al. [40], in a retrospective, single-center study, enrolled 487 patients treated by Paclitaxel DCB in the novo lesions between September 2009 and December 2013. According to the clinical presentation, patients were divided into stable coronary artery disease (CAD) (217 patients) and ACS (270 patients). There were no significant differences in baseline clinical characteristics between the two groups except for a higher rate of patients with anemia in the ACS group (32% in the ACS group and 20% in the stable CAD group, *p* = 0.005). At two years follow-ups, patients presenting with ACS, compared to those with stable CAD, showed higher total mortality (13.0 ± 1.8% and 9.3 ± 1.8%, respectively, *p* < 0.001) andMACE rates (19.3 ± 3.1% and 12.0 ± 2.0%, respectively, *p* = 0.012). Likewise, Tervo et al. [41], in a single retrospective study, enrolled 177 patients with stable CAD and 161 patients with ACS treated with paclitaxel-coated DCB between August 2014 and November 2018. The mean age of the patients was 71 ± 11 years, with 37% of diabetic patients and 55% considered at high bleeding risk. In the ACS group, 25% of the enrolled patients had ST-elevation myocardial infarction as a clinical presentation. The most common target vessel was the left anterior descending artery (33%), and in 64% of cases, two or more lesions were treated. At one year follow-up, total mortality after DCB-only PCI was 2.3% in the stable CAD Group and 12.6% in the ACS Group (*p* = 0.017), mainly driven by a higher cardiovascular death in the ACS Group (8.8%) compared to the stable CAD Group (1.1%, *p* = 0.0004).

Moreover, in the setting of ACS, in the 40 patients enrolled in the prospective, observational, DEB-AMI trial [24], a higher late lumen loss at 12 months has been observed in the DCB group (0.51 ± 0.59 mm) compared to DES (0.21 ± 0.32 mm, *p* < 0.01). Accordingly, the role of DCB PCI in the setting of ACS should be better evaluated in future and ongoing dedicated studies. 

## 5. Advantage of DCB in Large Vessel

Although the role of DCB in large coronary arteries (≥2.75 mm) is less settled and the safety compared to stent remains a concern, this setting is potentially appealing as stents, in general, are needed only in the initial phase of angioplasty in order to avoid acute vessel recoil and to treat flow limiting dissections. The use of DCB, releasing drugs without leaving metal behind, could offer some advantages, such as avoiding stent struts malapposition, especially in vessels with irregular walls, aneurismatic dilatation, or bifurcations. Moreover, the use of DCB could be preferred over DES implantation in high bleeding risk patients, allowing an early dual antiplatelet therapy de-escalation or fast P2Y12 discontinuation compared to DES even though the best duration of dual antiplatelet therapy after DCB PCI has not been defined. Besides, the use of DCB could also play a role in the setting of acute coronary syndromes, offering the advantages of restoring coronary flow and promoting vascular healing without the risks connected to inadequate stent sizing. Another important point is that DCB may be used in subsets of lesions where stents cannot be delivered or where stents do not perform well as long diffuse atherosclerotic lesions, as recently confirmed in a multi-center retrospective study enrolling 147 patients (over 80% with a lesion involving a large left anterior descending segment) that showed a similar 2-year target lesion failure between DCB and stent [32]. Similar data has been observed in the retrospective study performed by Leone et al. with a target lesion failure of 5.1% at 350 days follow-up [42]. Finally, in young patients, a strategy of “intervention without implantation” in the treatment of distal coronary stenosis might preserve the vessel for future bypass surgery interventions in case of proximal progression of the atherosclerotic disease.

## 6. Future and Ongoing Studies

Today, large, randomized studies comparing DCB and DES in large coronary arteries are lacking, but different studies such as the TRANSFORM II [43], the SELUTION [44], and the DCB-LVT (NCT05550233) are ongoing, enrolling thousands of patients and comparing DCB versus DES. These studies will give an answer to the possible extensive use of DCB in the treatment of large-vessel coronary disease. In particular, the TRANSFORM II trial (NCT04893291) will enroll more than 1800 patients with de novo coronary lesions with a diameter up to 3.5 mm by visual estimation and randomized to Sirolimus DCB only PCI or Everolimus DES PCI and followed up to 60 months follow-up. Notably, optimal lesion preparation in terms of predilation is encouraged for all study patients, as lesion pre-dilation using a step-by-step strategy has emerged as a pivotal determinant of DCB angioplasty in the treatment of coronary lesions. In current state-of-the-art practice, intracoronary imaging is becoming the standard of care, particularly in complex lesions, and in the TRANSFORM II trial, an intracoronary imaging sub-study will be carried out in centers experienced with optical coherence tomography at baseline and at nine months follow-up. 

The SELUTION Trial (NCT04859985) is an even larger multicenter randomized trial enrolling more than three thousand three hundred patients in 50 sites in Europe and Asia. Patients will be randomized to Sirolimus DCB only PCI or Everolimus DES PCI and followed up to 5 years. There is no limitation in the number of lesions to be treated, and de novo coronary lesions with diameters up to 5 mm can be included. Finally, the DCB-LVT (NCT05550233) will enroll 240 patients with large vessel coronary disease (diameter of the reference vessel is ≥3.0 mm) randomized to DCB-only PCI or DES PCI. Patients will be evaluated at 12 months follow-up by coronary angiography, and the primary endpoint is the late lumen loss.

## 7. Conclusions

Although there are no recommendations in the guidelines for the use of DCB in the “de novo” lesions, the literature evidence supports the use of DCB in de novo lesions for small vessel disease and suggests promising early results in the treatment of de novo lesions in large vessel disease. In general, DCB PCI in large vessels is feasible, but a careful selection of patients and adequate preparation of the target lesion are key criteria in the treatment of these lesions. While the evidence suggests the potential effectiveness of DCB, it is important to interpret the results with care, considering the variability in study dimensions and patient characteristics, and only the incoming large randomized clinical trials will clarify if the DCB technology will be a new option for the treatment of patients with large vessel coronary atherosclerosis.

## Figures and Tables

**Table 1:** Prospective and Retrospective Observational studies.

First Author	Year	Design	Patients or Lesions (*n*)		Clinical Presentation ACS, *n*		RVD, mm		Type DCB	FU (Months)	MACE, *n* (%)	
			DCB	Stent	DCB	Stent	DCB	Stent			DCB	Stent
Nijhoff et al. [24]	2015	P	40	49	40	49	2.8 ± 0.5	2.8 ± 0.5	Pac	12	5 (12.5)	1 (2)
Lu et al. [26]	2019	P	92	--	47	--	>2.75	--	Pac	11.4 ± 1.6	4 (4.3)	--
Yu et al. [25]	2019	R	200	--	183	--	3.2 ± 2.8	--	Pac	10	0 (0)	--
Rosenberg et al. [23]	2019	P	134	--	44	--	>2.75	--	Pac	9	7 (5.2)	--
Iwasaki et al. [27]	2021	R	69	88	2	2	3 ± 0.5	3 ± 0.4	Pac	12	6 (8.7)	4 (4.5)
Merinopoulos et al. [28]	2023	R	544	693	0	0	>2.75	>3.0	Pac	42	33 (5) TLR	32 (4.6) TLR
Nakamura et al. [29]	2023	R	73	81	0	0	3 ± 0.4	3.3 ± 0.4	Pac	14	8 (11)	21 (26)
Gunawarden et al. [30]	2023	R	41	107	28	57	3.8 ± 0.3	4.3 ± 0.6	Pac	33	5 (12.2)	23 (21.5)
Gitto et al. [31]	2023	R	139	139	0	0	3.1 ± 0.5	3.1 ± 0.4	Pac + Sir	24	4 (2.9)	24 (17.3)

ACS: acute coronary syndrome; DCB: Drug-coated Balloon; P: Prospective; Pac: Paclitaxel; R: Retrospective; RVD: reference vessel diameter; MACE: major adverse cardiovascular events (cardiovascular death, myocardial infarction, target lesion revascularization); Sir: Sirolimus.

**Table 2:** Randomized trials in large vessels.

First Author	Year	Total Patients, *n*		Clinical Presentation ACS *n* (%)		RVD, (mm)		Type DCB	Follow-Up Duration (months)	MACE *n*, (%)	
		DCB	Stent	DCB	Stent	DCB	Stent			DCB	Stent
Nishiyama et al. [32]	2016	27	33	27 (100)	33 (100)	2.9 ± 0.6	2.7 ± 0.6	Paclitaxel	8	0 (0)	2 (6.1)
Gobic et al. [33]	2017	38	37	38 (100)	37 (100)	2.6 ± 0.5	3.0 ± 0.5	Paclitaxel	6	2 (5.26)	2 (5.40)
Vos et al. [36]	2019	59	61	59 (100)	61 (100)	3.3 ± 0.5	3.2 ± 0.5	Paclitaxel	9	2 (3.39)	1 (1.63)
Rissanen et al. [34]	2019	102	106	47 (46)	49 (46)	2.5–4	2.5–4	Paclitaxel	9	1 (1.02)	15 (14.1)
Shin et al. [35]	2019	20	20	6 (30)	8 (40)	>2.8	>2.8	Paclitaxel	12	0 (0)	3 (15)
Yu X et al. [37]	2022	84	79	76 (91)	69 (87.4)	2.8 (2.5–3.3)	3.0 (2.7–3.4)	Paclitaxel	12	2 (2.38)	4 (5.06)
Wang et al. [38]	2022	92	92	92 (100)	92 (100)	3.31 ± 0.56	3.4 ± 0.5	Paclitaxel	12	5 (5.7)	6 (7.1)

ACS: acute coronary syndrome. DCB: Drug-coated Balloon; MACE: major adverse cardiovascular events; RVD: reference vessel diameter.

**Table 3:** Angiographic characteristics and target vessel revascularizations in randomized clinical trials.

Author	Total Patients, *n*		Lesion Location (LAD) (%)		*p*	Lesion Length (mm)		*p*	Late Lumen Loss		*p*	TLR (%)		*p*
	DCB	Stent	DCB	Stent		DCB	Stent		DCB	Stent		DCB	Stent	
Nishiyama [32]	27	33	44	52	0.699	16 ± 5	18 ± 7	0.241	0.25 ± 0.25	0.37 ± 0.40	0.185	0	6.1	0.193
Gobic [33]	38	37	NA	NA	-	NA	NA	-	−0.09 ± 0.09	0.10 ± 0.19	<0.05	NA	NA	-
Vos [36]	59	61	32	40	0.50	NA	NA	-	0.05	0.00	0.51	3	2	1.00
Rissanen [34]	102	106	40	38	0.94	NA	NA		NA	NA		0	6	0.015
Shin [35]	20	20	30	40	0.507	21 ± 3	19 ± 3	0.052	0.2 ± 0.3	1.2 ± 0.8	<0.001	0	15	0.106
Yu X [37]	84	79	57	44	0.101	18	20	0.202	−0.19 ± 0.49	0.03 ± 0.64	0.019	1.2	3.8	0.361
Wang [38]	92	92	55	57	0.836	31 ± 10	34 ± 13	0.095	0.24 ± 0.39	0.31 ± 0.38	0.266	2.3	2.4	1.00

DCB: Drug-coated Balloon; LAD: Left Anterior Descending; NA: Not Available; TLR: Target vessel revascularization.

## Data Availability

Data sharing is not applicable as no new data were created.
